# Albumin versus crystalloid and microcirculatory blood flow in ischaemia/reperfusion-induced spinal cord injury: a randomised porcine trial

**DOI:** 10.1016/j.bjao.2026.100860

**Published:** 2026-09-12

**Authors:** Lukas Steger, Vincent Umathum, Katharina Bock, Till Friedheim, Hans O. Pinnschmidt, Anne Schänzer, Celine Blaschek, Johanna Rehders, Tobias Pantel, Leonie Schulte-Uentrop, Bernd Saugel, Christoph R. Behem

**Affiliations:** 1 Department of Anaesthesiology, Center of Anaesthesiology and Intensive Care Medicine, University Medical Center Hamburg-Eppendorf, Hamburg, Germany; 2 Institute of Neuropathology, Justus-Liebig-University Giessen, Giessen, Germany; 3 Institute of Pathology and Molecular Pathology, German Armed Forces Hospital Ulm, Ulm, Germany; 4 Institute of Medical Biometry and Epidemiology, University Medical Center Hamburg-Eppendorf, Hamburg, Germany; 5 Department of Neurosurgery, University Medical Center Hamburg-Eppendorf, Hamburg, Germany

**Keywords:** albumin, microcirculation, reperfusion injury, spinal ischaemia

## Abstract

**Background:**

Spinal cord injury caused by ischaemia/reperfusion is a severe complication of aortic repair surgeries. Albumin may stabilise the endothelial barrier and preserve microcirculatory blood flow more effectively than crystalloids. This study tested whether albumin-based fluid therapy improves spinal cord microcirculation compared with crystalloid-based therapy in a porcine ischaemia/reperfusion model.

**Methods:**

Forty Duroc pigs were randomly assigned to receive albumin-based or crystalloid-based fluid therapy. Ischaemia/reperfusion injury was induced by 45 min of supracoeliac aortic cross-clamping. The primary outcome was spinal cord microcirculatory blood flow 4.5 h after the intervention. Secondary outcomes included spinal cord tissue oxygenation, somatosensory-evoked potentials, histopathology, and macrohaemodynamic variables.

**Results:**

Mean spinal cord microcirculatory blood flow (327.2 [95% confidence interval: 228.8–425.6] *vs* 276.9 [178.5–375.3] arbitrary unit [a.u.]) and tissue oxygenation (52.4 [39.4–65.5] *vs* 44.0 [31.8–56.2] %) did not differ significantly between albumin- and crystalloid-treated pigs. Somatosensory-evoked potential amplitudes of the tibial nerves (0.161 [0.082–0.241] *vs* 0.167 [0.082–0.253] μV) and the number of hypoxic neurons (2.69 [0.2–6.2] *vs* 2.58 [0.3–7.2] per mm^2^) were also similar in groups.

**Conclusions:**

Albumin-based fluid therapy did not improve spinal cord microcirculatory blood flow, oxygenation, or histopathological and functional outcomes compared with crystalloid-based therapy in this porcine model of ischaemia/reperfusion-induced spinal cord injury. This trial suggests testing whether albumin can improve spinal cord microcirculatory blood flow in trials in patients is not warranted.

**Clinical trial number and registry URL:**

AnimalStudyRegistry.org, https://doi.org/10.17590/asr.0000297

**Editor's key points**

• Question: Does albumin-*vs* crystalloid-based fluid therapy improve microcirculation in an experimental porcine animal model of ischaemia/reperfusion spinal cord injury?
• Findings: This randomised controlled animal trial showed no difference in microcirculatory blood flow or tissue oxygenation, and histopathological or functional outcomes in the spinal cord between the two groups.
• Meaning: A trial in humans aimed to improve microcirculation through albumin-based therapy in spinal cord injury does not seem warranted.

Spinal cord injury induced by ischaemia/reperfusion (I/R) is a substantial cause of perioperative morbidity and increased mortality in open and endovascular aortic repair surgery.^1^^,^^2^ A mechanistic understanding that integrates macro- and microcirculation is essential: systemic haemodynamics alone incompletely predict tissue perfusion, and macro–microcirculatory uncoupling is common during fluid resuscitation.^3^^,^^4^ Accordingly, effective prevention strategies must couple optimisation of spinal cord perfusion pressure with protection of the vulnerable microvasculature.^3^

Current guidelines emphasise bundled, multimodal protocols that preserve collateral blood flow, augment perfusion pressure and apply adjuncts.^2^^,^^5^ Cerebrospinal fluid drainage can increase spinal cord perfusion pressure through decreasing spinal fluid pressure, but its routine use warrants caution given procedure-related complications, e.g. postpuncture headache, subarachnoid haemorrhage, meningitis or neurological deficits.^6^^,^^7^ Hypothermia reduces metabolic demand and has long been used as part of integrated protection strategies during extensive aortic repair, but its side effects, e.g. impaired coagulation or surgical side infection, should be considered.^8^^,^^9^

Crystalloid solutions remain the mainstay of perioperative resuscitation because they are inexpensive, widely available, and effective for restoring circulating volume. However, they rapidly redistribute into the interstitial space, promoting tissue oedema, impairing oxygen diffusion and potentially aggravating spinal cord swelling.^10^ These limitations have driven interest in colloid-based approaches that may better preserve intravascular volume and endothelial stability.

Albumin contributes to plasma oncotic pressure and exerts additional protective effects on the endothelium. It also transports sphingosine-1-phosphate (S1P), a lipid mediator that stabilises endothelial junctions, preserves glycocalyx structure and reduces vascular permeability.^11^^,^^12^ Clinically, hypoalbuminaemia correlates with pulmonary oedema and adverse outcomes in critical illness and spinal cord injury (SCI), suggesting potential therapeutic relevance of albumin administration in vulnerable patients.^13^^,^^14^

We therefore conducted a randomised controlled porcine trial comparing albumin- and crystalloid-based fluid therapy during supracoeliac aortic cross-clamping. Specifically, we tested the primary hypothesis that albumin-based fluid therapy produces better spinal cord microcirculatory blood flow than crystalloid-based fluid therapy in an experimental porcine model of I/R-induced SCI.

## Methods

### Ethics

The Governmental Commission on the Care and Use of Animals of the City of Hamburg approved this trial (Reference number N042/2022). We provided animal care in compliance with the *Guide for the Care and Use of Laboratory Animals* (National Institutes of Health publication number 86–23, revised 2011) and all authors complied with the Animal Research: Reporting of In Vivo Experiments Guidelines.

### Animals

In total, 40 Duroc-race domestic pigs were included in this trial. Equally divided between male (50%) and female (50%) sex, animals were 4 months old and mean weight was 44.9 ± 3.7 kg when they were included. During a 14-day acclimatisation period, the animals were cared for by professional animal keepers and veterinarians of the university's animal research facility. The pigs were housed in an indoor animal facility, fed twice a day with water ad libitum and only separated from their group for a 12-h fasting period preceding the experiment. Before being transferred to the operating theatre, the pigs were analgo-sedated in their familiar environment.

### Trial design and protocol

**Fig 1:**
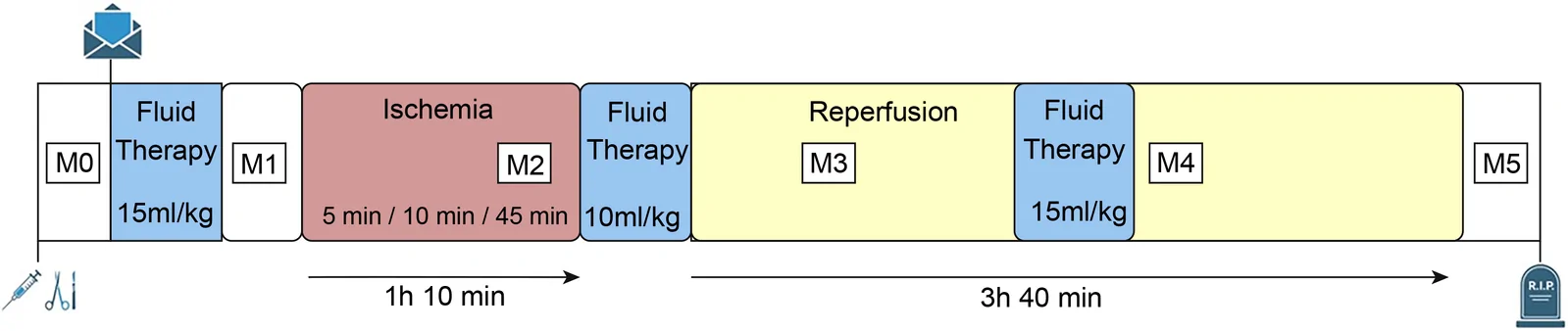
Trial protocol. After completion of anaesthesiologic and surgical procedures, baseline measurements were performed (M0). Next, we performed fluid therapy according to randomisation. Pigs assigned to albumin therapy received 15 ml kg^−1^ 5% albumin (Human Albumin 20% (Baxalta or Albiomin). Pigs assigned to crystalloid therapy received 15 ml kg ^−1^ crystalloids (Sterofundin ISO 1/1 E). After completion of fluid therapy, measurements were repeated (M1). Measurements were repeated at the end of the 45-min clamping interval (M2). We induced reperfusion by reopening the aortic clamp. Immediately after reperfusion, pigs received another 10 ml kg^−1^ of either albumin or crystalloids according to group randomisation. One h was allowed for haemodynamic stabilisation. Thereafter, measurements were repeated (M3); 2.5 h after ischaemia, we repeated fluid therapy with 15 ml kg^−1^ albumin or crystalloids according to group randomisation as previously described. After completion of fluid therapy, measurements were repeated (M4); 4.5 h after induction of I/R injury, final measurements were performed (M5). I/R, ischaemia/reperfusion; M1-5, measurement point 1–5; M1, fluid therapy before I/R; M2, ischaemia; M3, reperfusion; M4, fluid therapy after I/R; M5, 4.5 h after I/R.

Animals were randomly assigned to albumin-based fluid therapy or crystalloid-based fluid therapy. Group randomisation was performed using closed envelopes which were opened only after completion of anaesthesia induction, surgical procedures and measurement of baseline conditions. Severe complications, e.g. severe haemorrhage, before baseline measurements led to the exclusion of animals before randomisation. The trial protocol is shown in Figure 1.

### Anaesthesia and surgery

A detailed study protocol including surgical procedures and anaesthesia is described in the Supplementary Data. In brief, the trial was conducted under steady state general anaesthesia performed by continuous infusion of fentanyl (20 μg kg^−1^ h^−1^) and inhalation of sevoflurane (2.5 % expired concentration, Primus ®, Dräger Medical, Lübeck, Germany) after endotracheal intubation. Adequate anaesthesia depth was assessed through vital signs and EEG-monitoring (BIS Quatro ®, BIS LoC Channel, Covidien IIc, Mansfield, MA, USA). We targeted normothermia throughout the trial by convective warming and warming of fluids (Level 1 Hotline Fluid Warmer, Level 1 Technologies, Rockland, MA, USA).

After anaesthesia induction, we cannulated the right femoral artery and the right external jugular vein. A lumbar cerebrospinal fluid drainage was inserted and a cerebrospinal fluid pressure of 10 mm Hg maintained during the trial. Next, a hemilaminectomy for probe placement and a median sternotomy to enable supracoeliac aortic cross-clamping were performed (Supplementary Fig. 1). Moreover, the descending aorta was cannulated to obtain arterial pressure during aortic cross-clamping. Animals received 15 ml kg^−1^ albumin 5 % (Human Albumin 20%, Baxalta, Takeda GmbH, Konstanz, Germany, and Albiomin, Biotest Pharma GmbH, Dreieich, Germany) diluted in Sterofundin ISO 1/1 E, B. Braun, Melsungen, Germany) or crystalloid (Sterofundin ISO 1/1 E) volume loading therapy before aortic cross-clamping. After 5- and 10-min preconditioning intervals, a 45-min supracoeliac aortic cross-clamping interval was used to induce I/R injury. Immediately after reperfusion, pigs received another 10 ml kg^−1^ of albumin 5% or 10 ml kg^−1^ crystalloids according to the trial group. Another fluid bolus of 15 ml kg^−1^ was given 2.5 h after reperfusion. Final measurements were taken 4.5 h after initiation of the 45-min clamping interval. A mean arterial pressure (MAP) higher than 40 mm Hg was maintained throughout the trial by phenylephrine infusion.

All animals were euthanised under anaesthesia after the trial and spinal tissue was sampled for histopathological analysis.

### Outcomes

The primary outcome was spinal cord microcirculatory blood flow 4.5 h after I/R-induced SCI.

Secondary outcomes included spinal cord tissue oxygenation and function, hypoxic alterations at the sectional level, and macrohaemodynamic variables.

Spinal cord microcirculatory blood flow (flux [arbitrary unit (a.u.)]) and tissue oxygenation (%) were measured using a combined laser-Doppler/white light reflectance probe (CP1T-1000, Moor Instruments, Axminster, UK) placed on the exposed dura mater at the area of the thoraco-lumbar junction after hemilaminectomy. Representative images of hemilaminectomy and probe placement are presented in the supplements. Data recording and analysis were performed using the corresponding hard- and software (moorVMS-LDF®, moorVMS-OXY®, moor VMS-Research® Software, Moor Instruments). Mean values of 60-s data recording intervals were used for each measurement point.

Spinal cord function was assessed using somatosensory evoked potentials (SSEP). SSEPs were generated by stimulating tibial and median nerves of the lower and upper extremities to compare spinal cord function of affected and unaffected spinal cord regions. Alternating stimulation with constant current intensity was applied to the tibial and median nerves of each side by placing subdermal (SDN) needle electrodes (Inomed, Emmendingen, Germany) near the typical location of the nerves of the hindlimbs and forelimbs. Bilateral cortical N20-P25 potentials were recorded with Inomed SDN corkscrew electrodes at positions C3' and C4' for left and right median nerves and Cz for tibial nerves against a fronto-median reference electrode (Fz) according to the 10-20 system.^15^^,^^16^ Data were recorded and analysed using the dedicated hard- and software (Inomed Isis Xpert IOM System, Inomed). The mean value of amplitude (μV) and latency (ms) for both sides was used to determine spinal cord function.

Tissue samples from the lumbar spinal cord (L1–L3), each approximately 4 cm in length, were taken through laminectomy from pigs of both trial groups. Two representative transversal sections of this resected lumbar spinal cord were analysed. Tissue samples were fixed for at least 3 days in buffered 4 % formalin and processed for paraffin embedding using standard protocols. Next, 4-μm thick paraffin sections were stained with haematoxylin and eosin (H&E) (MEDITE Medical GmbH, Burgdorf, Germany). The sections were digitised using a Hamamatsu NanoZoomer 360 and evaluated with NDP.View.2. Artefacts such as folds and scratches were excluded from evaluation. The area of the grey matter was determined in mm^2^. All neurons that exhibited at least one sign of hypoxia were counted: eosinophilic neurons, so-called shrunken neurons, loss of Nissl substance, and neuronal degeneration.^17^ The number of neurons with hypoxic signs was related to the area of the grey matter and presented as the number of hypoxic neurons/mm^2^.

We also assessed macrocirculatory and metabolic variables at all measurement points using transcardiopulmonary thermodilution, and arterial and mixed venous blood gas analysis as previously described.^18^ In detail, we performed invasive arterial blood pressure measurement in combination with transcardiopulmonary thermodilution using a 5 Fr. thermistor tipped arterial catheter (PiCCO Femoral Catheter, Pulsion, Munich, Germany). We measured central venous pressure at the distal lumen of the 12 Fr 5-lumen central venous catheter. We recorded central venous pressure, femoral artery pressure and transcardiopulmonary thermodilution using the PiCCO2 monitoring system (PiCCO2®, Science edition, version 6.0, Pulsion). We zeroed all catheters to ambient air pressure and levelled them at right atrium for calibration before each measurement. We performed thermodilution using three consecutive injections of 15-ml cold saline at each measurement step. We performed data analysis off-line using PiCCO2 recordings. In addition, we performed arterial and mixed venous blood gas analysis for each measurement step (ABL90 FLEX blood gas analyser, Radiometer Medical ApS, Brønshøj, Denmark).

### Statistical analysis

Power analysis based on previous data revealed a minimum sample size of 15 for each group to detect a 30% difference of spinal cord microcirculatory blood flow between groups with an α of 0.05 and a power of 0.8.^19^ Serious complications, e.g. severe bleeding, pneumonia, stress-induced arrhythmias, etc., are common in large animal trials. Therefore, on the basis of our experience in previous trials, five additional animals per group were used to cover a potential dropout.

All authors approved the statistical plan before the start of the analysis. Distributions of interval-scaled data were assessed by visual inspections of histograms, given a small sample size may provide insufficient information for a conventional test for normality; right-skewed data were log-transformed before further parametric analyses.^20^^,^^21^ The dependent variables were subjected to general linear mixed model analyses, using the SPSS v. 28 routine GENLINMIXED (IBM, Armonk, NY, USA) for continuous data with an identity link function. For the primary outcome variable, models were specified with fixed effects for variable group baseline values. Random intercepts were fitted for animal and repeated measures within animals over time points were considered, applying an AR1 covariance matrix. We excluded random intercepts from models if the Hesse matrix was not definitely positive. Marginal means with 95% confidence interval (CI) were computed, followed by pairwise comparisons of group means through least significant difference tests. Statistical analyses were performed using the SPSS statistical software package 28 values were expressed as mean ± standard deviation. For statistical analysis of histopathological findings and creating the figures, GraphPad Prism (version 9.4.1, GraphPad Software, Boston, MA, USA) was used. For histopathological variables, unpaired two-tailed *t*-tests were used for descriptive group comparisons of continuous data; the test was considered appropriate owing to balanced group sizes and the robustness of parametric tests against moderate deviations from normality. Histopathological variables were expressed as mean (95% CI). Two-tailed *p*-values < 0.05 were considered as significant. All analyses of the raw data were blinded to the conducting investigators.

## Results

Two of the 40 pigs included in this trial had to be excluded before randomisation owing to injury to the heart during sternotomy leading to severe bleeding. Thus, 38 pigs, 19 per trial group, were randomly assigned and were eligible for evaluation. Baseline characteristics are listed in Table 1.

**Table 1:** Baseline characteristics for pigs assigned to albumin therapy compared with crystalloid therapy are presented below. All baseline data were collected after completion of anaesthetic and surgical procedures. Data are presented as mean [sd]. PCO_2_, partial pressure of carbon dioxide; ScvO_2_, central venous oxygen saturation; SSEP, somatosensory evoked potential.

Baseline characteristics	Albumin group	Crystalloid group
Spinal cord microcirculatory blood flow (a.u.)	263.9 [209.4]	169.2 [133.3]
Spinal cord tissue oxygenation (%)	45.1 [20.2]	34.5 [16.0]
Mean amplitude SSEP N. tibialis (μV)	0.315 [0.138]	0.394 [0.233]
Mean amplitude SSEP N. medianus (μV)	1.75 [1.04]	2.18 [1.26]
Mean latency SSEP N. tibialis (ms)	31.6 [7.00]	32.7 [4.05]
Mean latency SSEP N. medianus (ms)	19.2 [1.8]	19.7 [1.28]
Cardiac output (L/min)	2.92 [0.44]	3.28 [0.47]
Stroke volume (ml)	42.0 [10.7]	47.4 [9.40]
Heart rate (beats per min^−1^)	70.1 [13.0]	68.8 [11.2]
Systolic arterial pressure (mm Hg)	86.3 [16.3]	87.6 [17.8]
MAP (mm Hg)	59.9 [13.0]	62.3 [14.0]
Diastolic arterial pressure (mm Hg)	45.8 [10.7]	47.6 [12.1]
Cerebrospinal fluid pressure (mm Hg)	13.4 [2.36]	12.2 [4.36]
Spinal cord perfusion pressure (mm Hg)	46.5 [20.2]	49.7 [13.9]
Central venous pressure (mm Hg)	4.88 [1.57]	5.12 [3.00]
Pulse pressure variation (%)	11.4 [4.03]	10.8 [3.43]
Global end-diastolic volume (ml)	534.9 [77.6]	581.7 [76.9]
Extravascular lung water (ml)	368.2 [41.8]	400.2 [66.1]
Lactate (mmol/L)	2.32 [0.65]	2.43 [0.86]
Base excess (mmol/L)	7.79 [2.62]	8.51 [2.52]
ScvO_2_ (%)	52.4 [15.1]	56.6 [15.6]
Venous-to-arterial PCO_2_ gap (kPa)	1.60 [0.51]	1.44 [0.59]

**Fig 2:**
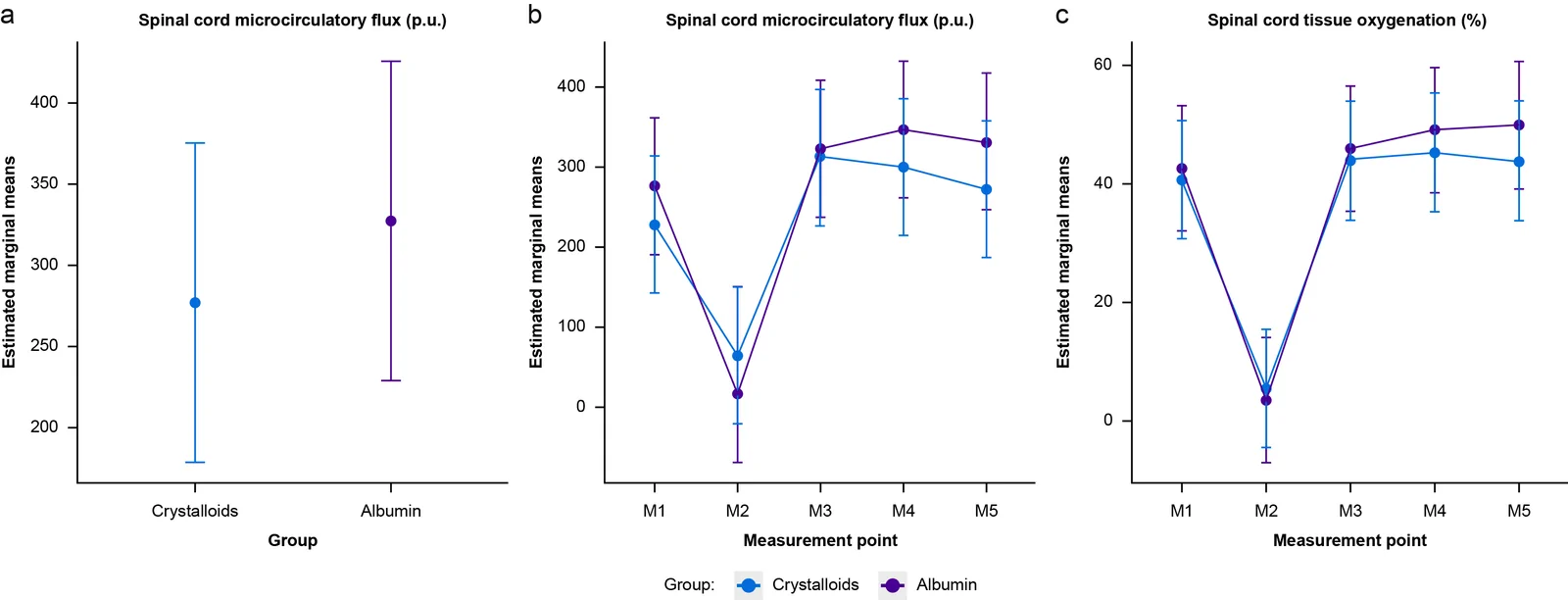
Spinal cord microcirculation. Comparison of the primary outcome spinal cord microcirculatory blood flow at the end point (A) and its trajectory (B), and tissue oxygenation (C) in pigs assigned to albumin therapy and to crystalloid therapy for the measurement points of the protocol. Because baseline values were included as fixed effect to the statistical model, baseline values are not given as outcome parameters. Data are presented as mixed model estimated marginal means over final measurement points in (A) and per measurement point in (B) and (C) for microcirculatory blood flow (a.u.) and tissue oxygenation (%); vertical lines with upper and lower bars represent 95% CI. I/R, ischaemia/reperfusion; M1, fluid therapy before I/R; M2, ischaemia; M3, reperfusion; M4, fluid therapy after I/R; M5, 4.5 h after I/R.

There were no clinically meaningful or statistically significant differences in mean spinal cord microcirculatory blood flow (327.2 [95% CI: 228.8–425.6] *vs* 276.9 [95% CI: 178.5–375.3] a.u., *p* = 0.476; Fig. 2A and B) between pigs assigned to albumin-based and those receiving crystalloid-based fluid therapy. Spinal cord tissue oxygenation (52.4% [95% CI: 39.4–65.5] *vs* 44.0% [95% CI: 31.8–56.2], p = .349; Fig. 2C) was also similar in pigs assigned to albumin-based and those to crystalloid-based fluid therapy. Because baseline values were included as fixed effect to the statistical model, baseline values are not given as outcome parameters. Ischaemia markedly reduced spinal cord microcirculatory blood flow and tissue oxygenation.

**Fig 3:**
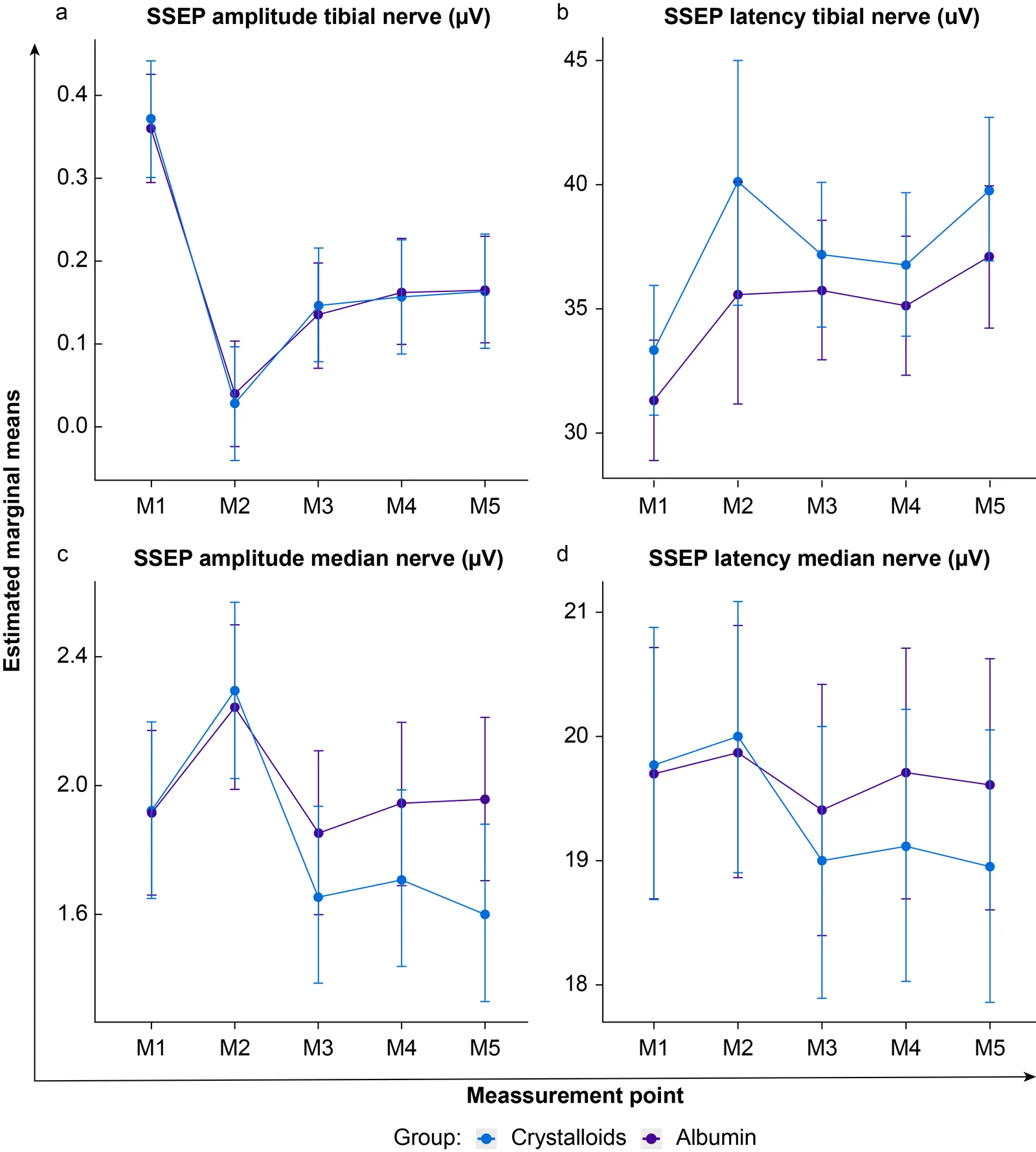
Spinal cord function. Comparison of SSEP amplitudes and latencies for the tibial (A, B) and median (C, D) nerves in pigs assigned to albumin therapy and to crystalloid therapy for the measurement points of the protocol. Because baseline values were included as fixed effect to the statistical model, baseline values are not given as outcome parameters. Data are presented as mixed model estimated marginal means of mean amplitudes (μV), vertical lines with upper and lower bar representing 95 % CI. I/R, ischaemia/reperfusion; M1, fluid therapy before I/R, M2, ischaemia; M3, reperfusion; M4, fluid therapy after I/R, M5, 4.5 h after I/R.

There were no significant differences in the function of I/R-affected spinal cord regions at the experimental end point between pigs assigned to albumin-based and those to crystalloid-based fluid therapy regarding the SSEPs. Both the amplitudes of the tibial SSEPs (0.161 [95% CI: 0.082–0.241] *vs* 0.167 [95% CI: 0.082–0.253] μV, *p* = 0.917; Fig. 3A] and the tibial SSEP latencies [37.6 [95% CI: 32.9–42.4] *vs* 40.2 [95% CI: 35.5–44.8 ms], *p* = 0.446; Fig. 3B) were similar in the groups. As expected, spinal cord regions unaffected by I/R injury produced similar SSEP amplitudes (2.00 [95% CI: 1.67–2.33] *vs* 1.63 [95% CI: 1.27–1.98] μV, *p* = 0.136; Fig. 3C) and SSEP latencies (19.6 [95% CI: 18.7–20.5] *vs* 18.9 [95% CI: 18.0–19.9] ms, *p* = 0.326; Fig. 3D) of the median nerve.

Because baseline values were included as fixed effect to the statistical model, baseline values are not given as outcome parameters. Ischaemia markedly reduced sensory evoked potential (SEP) amplitudes of the tibial nerve, whereas SEP amplitudes of the median nerve increased.

**Fig 4:**
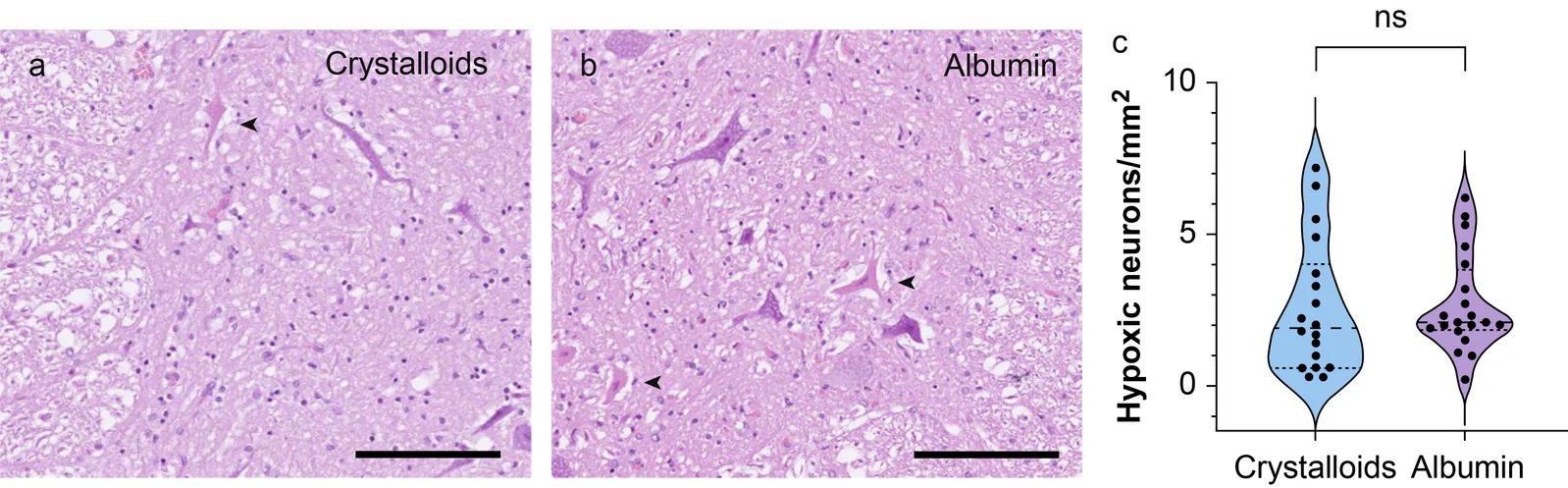
Histomorphological analysis of hypoxia-induced pathology. Representative images at H&E-stained section of the spinal cord of pigs after interventional I/R. Histology of pigs treated with crystalloid (A) and albumin agents (B). After I/R intervention, hypoxia-induced changes, such as moderate vacuolation and eosinophilic shrunken neurons (arrowheads), are visible. Analysis of hypoxic neurons per mm^2^ does not show significant differences between pigs treated with crystalloids and with albumin (*p* = 0.851, C). Albumin, pigs assigned to albumin therapy; Crystalloids, pigs assigned to crystalloid therapy; I/R, ischaemia/reperfusion; ns, non-significant. Bar: 100 μm.

No obvious hypoxic pathology at the spinal cord samples such as greyish discolouration, bleeding, or softening was observed. Hypoxic changes at H&E-stained sections were observed in the spinal cord of pigs assigned to albumin or crystalloid therapy after I/R. Specifically, a microcystic vacuolisation of the grey matter with hypoxic neurons, predominantly affecting the neurons of the anterior horn, was observed. Morphometric analysis of the density of hypoxic neurons revealed no significant differences between pigs treated with albumin and pigs treated with crystalloid (2.69 [95% CI: 0.2–6.2] *vs* 2.58 [95% CI: 0.3–7.2] hypoxic neurons per mm^2^, *p* = 0.851; Fig. 4). In the white matter, microcystic vacuolisation was observed. Immunolabelling using antibody against neurofilament showed axonal loosening and some axonal swelling was observed, consistent with early mild hypoxia-induced changes (Supplementary Fig. 2).

Pigs assigned to albumin-based fluid therapy had significantly higher values of cardiac output, stroke volume, systolic, mean and diastolic arterial pressure, spinal cord perfusion pressure, central venous pressure, global end-diastolic volume, and central venous oxygen saturation and significantly lower values of pulse pressure variation and venous-to-arterial partial pressure of carbon dioxide gap than did pigs assigned to crystalloid-based fluid therapy (Supplementary Fig. 3). There were no significant differences of lactate levels, base excess, heart rate, extravascular lung water and cerebrospinal fluid pressure between groups (Table 2).

**Table 2:** **Comparison of macrohaemodynamic and metabolic variables in pigs assigned to albumin therapy and to crystalloid therapy 4.5 h after I/R.** Data are given as mixed model estimated marginal means (95 % CI). For all variables, *p-*values for pairwise comparison of groups are given. CI, confidence interval; PCO_2_, partial pressure of carbon dioxide; I/R, ischaemia/reperfusion; ScvO_2_, central venous oxygen saturation.

Macrohaemodynamic and metabolic variables	Albumin group	Crystalloid group	*p*-value
Cardiac output (L/min)	4.50 (4.07–4.93)	3.12 (2.68–3.57)	***p* < 0.001**
Stroke volume (ml)	53.4 (49.6–57.2)	37.5 (33.6–41.4)	***p* < 0.001**
Heart rate (beats min^−1^)	83.4 (74.0–92.9)	88.2 (78.5–97.9)	*p* = 0.479
Systolic arterial pressure (mm Hg)	95.2 (87.2–103.2)	76.2 (67.9–84.4)	***p* = 0.002**
MAP (mm Hg)	65.8 (59.4–72.2)	52.2 (45.6–58.7)	***p* = 0.005**
Diastolic arterial pressure (mm Hg)	47.3 (41.9–52.7)	37.8 (32.3–43.3)	***p* = 0.017**
Cerebrospinal fluid pressure (mm Hg)	15.4 (14.1–16.8)	14.3 (13.0–15.6)	*p* = 0.227
Spinal cord perfusion pressure (mm Hg)	50.1 (43.1–57.2)	37.7 (30.4–44.9)	***p* = 0.017**
Central venous pressure (mm Hg)	5.95 (5.50–6.40)	4.45 (4.00–0.89)	***p* < 0.001**
Pulse pressure variation (%)	7.69 (5.36–10.0)	14.0 (11.6–16.4)	***p* = 0.001**
Global end-diastolic volume (ml)	573.0 (536.6–609.4)	486.9 (449.4–524.4)	***p* = 0.002**
Extravascular lung water (ml)	415.6 (391.9–439.4)	423.2 (398.7–447.8)	*p* = 0.660
Lactate (mmol/L)	3.65 (2.96–4.35)	4.03 (3.36–4.71)	*p* = 0.432
Base excess (mmol/L)	5.17 (3.71–6.64)	3.88 (2.46–5.31)	*p* = 0.212
ScvO_2_ (%)	72.8 (66.0–79.6)	51.7 (45.0–58.3)	***p* < 0.001**
Venous-to-arterial PCO_2_ gap (kPa)	0.91 (0.68–1.14)	1.47 (1.24–1.69)	***p* = 0.001**

## Discussion

In our trial, albumin-based fluid therapy produced similar spinal cord microcirculatory blood flow to crystalloid-based fluid therapy in an experimental porcine model of I/R-induced SCI—although albumin-based fluid therapy yielded higher cardiac output and arterial and spinal cord perfusion pressure than did crystalloid-based fluid therapy.

The increase in blood pressure and therefore spinal cord perfusion pressure we observed are consistent with findings of trials investigating albumin use in patients with critical illness**.**^22^ However, improved macrohaemodynamics do not necessarily lead to improved microcirculation and clinical outcomes.^23^ The uncoupling of the macrocirculation and the microcirculation can cause inadequate organ perfusion, a phenomenon previously described in both patients with critical illness and those having surgery.^24^ However, the restoration of microcirculatory flow to baseline values, rather than hyperaemia, may represent a favourable outcome, given postreperfusion hyperaemia correlates with adverse outcomes.^25^ Our measurement time points were strategically selected to capture acute I/R dynamics rather than delayed microcirculatory dysfunction.

An *in vivo* model in rats indicated that albumin mitigates ischaemia–reperfusion-induced endothelial and glycocalyx injury *in vivo* through S1P-dependent mechanisms.^26^ Although the previously described glycocalyx-preserving effects of albumin may have occurred, albumin-based fluid therapy was not able to overcome the multifactorial uncoupling of macro- and microcirculation. We did not include glycocalyx degradation markers in our trial because methodologically comparable trials failed to show correlations between glycocalyx markers and clinical outcomes in major cardiac and abdominal surgery with short-term follow-up.^27^ Meta-analyses that have proposed prognostic value of glycocalyx biomarkers in patients with sepsis have predominantly involved older patients with multiple comorbidities.^28^

In addition to *in vivo* measurements of spinal perfusion, we evaluated functional outcomes of the fluid therapy regimens. We assessed SCI through SSEPs in upper and lower extremities. An SCI model has shown that SSEPs in humans are comparable to those of pigs.^29^ As expected, upper extremity SSEPs remained nonpathological with an increase in amplitude, which may be attributed to various reasons in I/R injury, whereas lower-extremity SSEPs showed decreased amplitude during ischaemia.^30^ An animal model of cerebral microcirculation in rats has indicated that fluid therapy combined with vasopressors was capable of preserving SSEPs in a haemorrhagic shock model.^31^ In our trial comparing two different fluid therapy regimens, albumin-based fluid therapy was not able to improve spinal cord function assessed by SSEPs compared with crystalloid-based fluid therapy, suggesting no clinical intraoperative benefit in the prevention of paralysis. Although mean arterial and spinal cord perfusion pressure in the albumin-based fluid therapy group was in ranges generally considered safe, functional impairment was not prevented. This might suggest other independent risk factors for SCI in I/R during aortic surgery.

Owing to its limited glycogen storage capacity, anaerobic metabolism in the central nervous system is severely restricted, unlike in skeletal muscle.^32^ Irreversible neuronal cell damage can occur after 7 min of complete circulatory arrest followed by reperfusion during hypoxic metabolism. In cases of incomplete blood flow, the revival interval after reperfusion can extend up to 3–6 h.^33^ Our histological analysis shows that spinal cord tissue of pigs subjected to experimental I/R exhibits significant hypoxia-induced changes, including microcystic vacuolisation and eosinophilic or shrunken neurons in the grey matter. The location of these changes aligns with observations in preclinical pig models, with the most severe damage occurring in neurons in the anterior and posterior horns of the spinal cord. White matter damage, such as myelin degeneration, was observed in 17% of the examined pig models. Our findings showed early microscopic hypoxia-induced changes in the white matter.^34^

There were no significant differences in the number of hypoxic neurons between pigs assigned to albumin-based and those assigned to crystalloid-based fluid therapy. These findings reflect our observations regarding the microcirculation, which also was similar in the two groups.

Regarding our methodological approach, several considerations warrant discussion. Our group previously validated laser-Doppler flowmetry against microsphere methods, the reference standard for microvascular perfusion.^35^ In contrast to microspheres, Laser-Doppler provides continuous monitoring and simultaneous tissue oxygenation measurement. To minimise measurement bias, we controlled for physiological confounders through standardised environmental conditions: constant lighting, stable room temperature and maintained normothermia. The 45-min aortic cross-clamping duration reflects our group's validated I/R injury model and is widely adopted in experimental I/R research.^36^, ^37^, ^38^ This interval corresponds to the clinical threshold of 30–40 min identified as critical for I/R injury during aortic repair.^39^ Furthermore, we would like to elaborate two considerations concerning the haemodynamic optimisation strategy. We used fixed-volume fluid administration rather than goal-directed fluid therapy based on stroke volume or pulse pressure variation. Our previous work revealed that colloids exhibit prolonged haemodynamic effects compared with crystalloids, differentially influencing dynamic preload parameters and potentially necessitating disparate fluid volumes to achieve the same end points.^18^ We believe that volume differences between groups would confound assessment of albumin's intrinsic glycocalyx-stabilising properties. Furthermore, we decided to withhold catecholamines until MAP was lower than 40 mm Hg to mitigate any catecholamine-mediated effects. The absence of superiority under these conditions strengthens our conclusion that albumin confers no microcirculatory advantage in I/R-induced SCI.

Current guidelines on fluid resuscitation restrict the use of albumin in traumatic brain injury because of proposed adverse neurological outcomes.^40^ In this context, the widespread use of albumin in cardiac anaesthesia presents a grey area in clinical decision-making in regard to supracoeliac aortic cross-clamping with a high risk of SCI.^41^ Our trial aiming to investigate albumin-based fluid therapy to preserve microcirculation and therefore spinal function and to improve neurological outcomes, despite showing no meaningful difference in microcirculation, did not reveal immediate perioperative negative neurological outcomes associated with albumin use.

This trial investigates the use of albumin-based fluid therapy in the perioperative period with a limited follow-up. Therefore, we cannot draw conclusions regarding microvasculature-preserving properties of albumin which may have long-term implications for vascular permeability, tissue oedema, and consequent microvasculatory compromise.

### Conclusion

Albumin-based fluid therapy did not yield better spinal cord microcirculatory blood flow than did crystalloid-based fluid therapy in an experimental porcine animal model of ischaemia/reperfusion induced spinal cord injury. Our results thus suggest that testing whether albumin can improve spinal cord microcirculatory blood flow in trials in patients is not warranted.

## Authors’ contributions

Conceptualisation: LS, VU, KB, TF, LS-U

Investigation: LS, KB, TF, CB, TR, CRB, JR

Formal analysis: LS, VU, HOP, CB, JR

Validation: LS, VU, HOP, BS, LS-U

Writing—original draft: LS, VU, KB, TF, VU

Writing—review and editing: LS, VU, KB, TF, VU, KB, TP, HOP, AS, LS-U, BS

Methods: KB, TF, TP, CRB

Morphometrical and data analysis: AS

Supervision: AS

Discussion of data: AS

Statistical conceptualisation: HOP

Writing—review and editing supervision: BS, CRB

Writing: BS

Resources: BS, CRB

All authors read and approved the final manuscript.

## Declaration of generative AI and AI-assisted technologies in the writing process

The authors declare that all data concerning the trial outcome are available within the article and the supplementary files. Raw data are available on reasonable request. During the preparation of this manuscript, the authors used the ChatGPT Default model (version GPT-3.5) developed by OpenAI (San Francisco, CA, USA) as an artificial intelligence-based tool to enhance clarity, coherence, and overall quality while maintaining the original creative and intellectual contributions of the authors. After using this tool, the authors reviewed and edited the content as necessary, taking full responsibility for the content of the article.

## Funding

This trial was funded by a restricted research grant (’Grifols Albus Award 2021’) from Grifols (Barcelona, Spain). The grant solely provided funding for the trial and did not influence trial design or methods.

## Declarations of interest

BS is a consultant for Edwards Lifesciences (Irvine, CA, USA), Philips North America (Cambridge, MA, USA), GE Healthcare (Chicago, IL, USA), Vygon (Aachen, Germany), Masimo (Neuchâtel, Switzerland), Retia Medical (Valhalla, NY, USA), Maquet Critical Care (Solna, Sweden), Pulsion Medical Systems (Feldkirchen, Germany), Dynocardia (Cambridge, MA, USA) and RDS (Strasbourg, France). BS has received institutional restricted research grants from Edwards Lifesciences, Baxter (Deerfield, IL, USA), GE Healthcare, Masimo, Philips Medizin Systeme Böblingen (Böblingen, Germany), CNSystems Medizintechnik (Graz, Austria), Pulsion Medical Systems, Vygon, Retia Medical and Osypka Medical (Berlin, Germany). BS has received honoraria for giving lectures from Edwards Lifesciences, Philips Medizin Systeme Böblingen, Baxter, GE Healthcare, Masimo, CNSystems Medizintechnik, Getinge (Gothenburg, Sweden), Pulsion Medical Systems, Vygon and Ratiopharm (Ulm, Germany). BS is an editor of the *British Journal of Anaesthesia*.
