# Supplementary material for: Albumin versus crystalloid and microcirculatory blood flow in ischaemia/reperfusion-induced spinal cord injury: a randomised porcine trial
Source: BJA Open. 2026 Sep 12;20:100860. doi: 10.1016/j.bjao.2026.100860 (PMC13584136; doi:10.1016/j.bjao.2026.100860)
Supplement: Supplementary file 1 [file mmc1.docx]

**Supplements**

## Study Protocol

All pigs received a baseline infusion rate of 10 ml kg^-1^ h^-1^ balanced isotonic crystalloids iv (Sterofundin ® ISO 1/1 E, B. Braun ®, Melsungen, Germany) to compensate for fluid losses during anesthesia and surgery. The randomized therapy was the type of fluid given before and after ischemia/reperfusion-induced spinal cord injury. Pigs assigned to albumin therapy received 15 ml kg^-1^ albumin 5 % (Human Albumin 20% (Baxalta ®, Takeda GmbH, Konstanz Germany, and Albiomin®, Biotest Pharma GmbH, Dreieich, Germany) diluted in Sterofundin ® ISO 1/1 E, B. Braun ®, Melsungen, Germany) using a maximum flow rate of 1200 ml h^-1^ (Infusomat ® Space, Braun ®, Melsungen, Germany) before ischemia/reperfusion-induced spinal cord injury. Pigs assigned to crystalloid therapy received 15 ml kg^-1^ of crystalloids (Sterofundin ® ISO 1/1 E, B. Braun ®, Melsungen, Germany) using a maximum flow rate of 1200 ml h^-1^ (Infusomat ® Space, Braun ®, Melsungen, Germany) before ischemia/reperfusion-induced spinal cord injury. Thereafter, we induced ischemia/reperfusion by a total of 60 minutes of supra-celiac aortic-cross-clamping, using preconditioning effects of 5- and 10-minute intervals prior a 45-minute aortic-cross-clamping interval to improve survival of animals during the trial period. We performed manual inflow occlusion of the inferior vena cava to prevent an increase of mean arterial pressure > 100 mmHg during aortic-cross-clamping. We induced reperfusion by re-opening of the aortic clamp. Immediately after reperfusion, pigs assigned to albumin therapy received another 10 ml kg^-1^ of albumin 5 %, while pigs assigned to crystalloid therapy received another 10 ml kg^-1^ of crystalloids. 2.5 h after reperfusion, pigs assigned to albumin therapy received another 15 ml kg^-1^ of albumin 5 %, while pigs assigned crystalloid therapy received another 15 ml kg^-1^ of crystalloids. Animals received phenylephrine to maintain a mean arterial pressure above 40 mmHg following reperfusion using a pre-defined protocol (100 μg bolus administered at reperfusion followed by continuous administration of 1.0 μg kg min^-1^ iv, reduced in μg kg^-1^ min^-1^ iv steps as needed). Cerebrospinal fluid drainage was performed to maintain a cerebrospinal fluid pressure of 10 mmHg. Induction of ischemia/reperfusion-induced spinal cord injury was defined as the start of the 45-minute aortic-cross-clamping interval. Measurements were performed at baseline (BASELINE, M0), following fluid therapy before ischemia/reperfusion (FT_PRE, M1), during ischemia (ISC, M2), following reperfusion (REP, M3), and following fluid therapy after ischemia/reperfusion (FT_POST, M4). Final measurements were performed 4.5 hours after induction of ischemia/reperfusion-induced spinal cord injury (I/R 4.5h, M5). At all measurement points microcirculatory blood flow and tissue oxygenation of the spinal cord as well as somatosensory evoked potentials to evaluate spinal cord function, were assessed. Additionally, macrocirculatory variables were measured.

## Anesthesia and Surgical Procedures

Anesthesia and surgical procedures were performed as previously described (Behem et al., 2022). We performed a tracheal intubation in all pigs after ensuring proper depth of analgo-sedation induced by intramuscular injections of ketamine 25 mg kg^-1^ and xylazine 2.5 mg kg^-1^. Thereafter, general anesthesia was accomplished by continuous infusion of fentanyl (20 µg kg^-1^ h^-1^ iv) and inhalation of sevoflurane (2.5 % expired concentration, Primus ®, Dräger Medical, Lübeck, Germany). We assessed adequacy of anesthesia by careful observation of vital signs and ventilation parameters as well as by absence of any movements during the entire protocol with special attendance to phases of surgical stimulus. In addition, EEG-monitoring was performed to evaluate the depth of anesthesia (BIS Quatro ®, BIS LoC Channel, Covidien IIc, Mansfield, MA, USA). Additional bolus doses of fentanyl (50 µg iv) were given if there was any indication of pain or distress. Pancuronium (0.15 mg kg^-1^ initial dose, 0.15 mg kg^-1^ repetition dose before sternotomy iv) was given for tracheal intubation and sternotomy only. A volume-controlled ventilation was established using tidal volumes of 10 mL kg^-1^ and a positive end-expiratory pressure of 10 cmH2O and ventilator frequency was adjusted to maintain an end-expiratory carbon dioxide tension (etCO2) of 35-45 mmHg (Primus ®, Dräger Medical, Lübeck, Germany). Unfractionated heparin (5.000 I.U. iv, followed by 10 I.U. kg^-1^ h^-1^ iv) was given after placement of vascular catheters to prevent blood clotting and thrombus formation. Clindamycin (300 mg iv initially, repetitive dose of 300 mg iv after 6 h) was given as antibiotic prophylaxis and prednisolone (bolus iv injection, 250 mg) was given to limit surgical induced inflammation during preparation. The entire experimental procedure and handling of pigs was supervised by the responsible veterinarian.

Following tracheal intubation, the right femoral artery and the right external jugular vein were cannulated by use of sonographic guidance. Next, a hemilaminectomy for placement of the spinal cord laser-Doppler/tissue oxygenation probe was performed at the thoraco-lumbar junction as shown in supplement figure 1. A lumbar cerebrospinal fluid drainage was inserted by transcutaneous punction of the intrathecal space caudal of the area of hemilaminectomy. Next, a median sternotomy was performed. The base of the left lung was mobilized, and the descending aorta was exposed by gentle retraction of the left lung ensuring undisturbed ventilation and avoiding trauma to the left lung. A vessel loop was placed around the thoracic descending aorta to mark the area of supra-celiac aortic-cross-clamping. In addition, the descending aorta was cannulated via a vascular catheter proximal of the cross-clamping area for measurement of aortic pressure during aortic cross clamping.

Somatosensory evoked potentials were part of each measurement point. They were recorded at 200 µs pulse width, with a frequency of 4.7 Hz and a filter of 5 – 600 Hz. An average of 200 sweeps was analyzed.

## Euthanasia and organ harvesting

After completion of the study protocol, all pigs were sacrificed during deep anesthesia by injection of Pentobarbital (50-60 mg kg^-1^) and spinal cord tissue was harvested for histopathological evaluation.

**Supplement Figure 1: Surgical Approach**

Spinal cord exposed by hemilaminectomy (dotted circle) (A) and placement of the laser-Doppler/tissue oxygenation probe (dotted circle) (B)

**Supplemental Figure 2: Neurofilament staining of the white matter.**

White matter from pigs not exposed to ischemic conditions shows no early signs of hypoxia when stained with an antibody against neurofilament (A). In contrast, white matter from pigs subjected to aortic clamping exhibits axonal swellings and occasional spheroid bodies (arrows) (B). Scale bar: 100 µm.

**Supplemental Figure 3: Selected hemodynamic outcome parameters**

Animals assigned to albumin-based fluid therapy therapy had significantly higher values of systolic (A), mean (B) and diastolic (C) arterial as well as central venous (D) pressure. Cardiac output (F), spinal cord perfusion pressure (G), stroke volume (H), global end-diastolic volume (K), and central venous oxygen saturation (O) were also significantly higher. Pulse pressure variation (J) and venous-to-arterial PCO_2_ gap (P) were significantly lower in pigs receiving albumin-based therapy. There was no difference in heart rate (E), stroke volume (I), extra-vascular lung water (L), lactate (M) and base excess (N). Measurements acquired through thermodilution were not available during aortic cross-clamping.
